# Examining Sociocultural Influences on Breastfeeding Attitudes Among Syrian and Hungarian Female Students

**DOI:** 10.3390/nu17020288

**Published:** 2025-01-14

**Authors:** Manar Al Kamsheh, Krisztina Antónia Bornemissza, Alexandra Zimonyi-Bakó, Helga Judit Feith

**Affiliations:** 1 Health Sciences Division, Doctoral College, Semmelweis University, 1085 Budapest, Hungary; manarkamsheh@gmail.com; 2 Faculty of Humanities and Social Sciences, Pázmány Péter Catholic University, 1088 Budapest, Hungary; bornemissza.krisztina.job@gmail.com; 3 Institute of Languages for Specific Purposes, Semmelweis University, 1091 Budapest, Hungary; bako.alexandra@semmelweis.hu; 4 Department of Social Sciences, Faculty of Health Sciences, Semmelweis University, 1085 Budapest, Hungary

**Keywords:** breastfeeding, bottle-feeding, Syria, Hungary, female students, sociocultural factors, behaviours

## Abstract

Background: Breastfeeding in Syria is a common practice supported by social norms, family traditions, and cultural values. In Hungary, recent statistics show that exclusive breastfeeding is significantly lower than the recommendation of the World Health Organization. Understanding the perspectives of educated young ladies is crucial for discovering the difficulties of breastfeeding practices within Syrian–Hungarian societies. This study explores the sociocultural factors and their impact on breastfeeding behaviours among female students in Syria and Hungary. Methods: A comprehensive, multi-section questionnaire was administered to 317 students from Damascus University and 303 students from Hungarian universities, designed to assess breastfeeding behaviours evaluated through The Breastfeeding Behaviour Questionnaire (BBQ). Results: The results in both societies showed remarkable awareness and understanding among participants regarding breastfeeding. Traditions and culture affect Syrian society more than Hungarian society; the two societies have restricted responses toward breastfeeding in public and different reactions to breastfeeding in front of males or females. Most students disagree with preferring formula feeding to breastfeeding when it is related to the family or the husband’s desire only. At the same time, agreement with choosing the bottle when the mother returns to work instead of exclusively breastfeeding is valued differently in the two societies. Conclusions: This study elucidates the essential the sociocultural factors influencing breastfeeding attitudes among Syrian and Hungarian female students, highlighting the need for culturally suitable strategies to improve breastfeeding practices in both countries.

## 1. Introduction

Breastfeeding (BF) is one of the most effective methods for ensuring child health and survival. WHO and UNICEF continually promote BF since it is the best nutrition for infants and children [1,2].

BF is widely recognised as the most efficient public health measure for decreasing mortality among children under five years [3]. Extended BF results in decreased infectious morbidity and mortality, fewer dental malocclusions, and increased intellect compared to shorter BF durations or no BF at all [4]. In addition, BF improves babies’ immune systems and can protect them against chronic diseases like diabetes and obesity [2]. The first step in managing chronic disease should be to emphasise BF, especially an extended duration of BF after exclusive BF for the first six months of life [5]. Exclusive BF has been positively associated with both flexibility and lower-body strength [6], as such adolescents have performed better in standing long jump tests, regardless of their fat mass, fat-free mass, or height [7].

BF has benefits not only for infants but also for mothers; BF protects the lactating mother from osteoporosis, breast cancer, ovarian cancer, and type 2 diabetes [4,8]. Additionally, it reduces the risk of postpartum depression and haemorrhage [9], and it promotes weight loss, natural contraception, and stress reduction [5]. Moreover, lactating women have been reported to seek medical care less frequently, experiencing fewer respiratory, cardiocirculatory, and gastrointestinal diseases and fewer emotional problems and symptoms [10]. Since the mother’s mental condition improves automatically when her baby’s health increases, and given the health benefits of BF for the baby, BF is key to maternal mental health [11].

BF practices differ significantly across regions, with some areas encountering more difficulties than others. BF rates vary widely between low- and middle-income and high-income countries. In low- and middle-income countries, only 4% of infants are not breastfed, while 21% of babies never receive breastmilk in high-income countries [9].

Despite the recommendations from WHO and UNICEF, the rates of exclusive BF remain very low worldwide [4], including Arab countries [12,13]; only 37% of infants under six months old are exclusively breastfed worldwide [1,4]. Moreover, about 35% of infants are exclusively breastfed in the Middle East [2]. The prevalence of exclusive BF in Hungary was found to be 44% at 6 months, among the highest in the region, as indicated by research on breastfeeding practices in the WHO European Region [14], but it has decreased in a few years to 35.16% [15], while exclusive BF until five months in Syria is only 29%, according to the UNICEF database [16]. Generally, in both cultures, breastfeeding is generally seen as a natural and important way to nourish a baby, with many women acknowledging its health benefits for both the mother and child [17,18].

Levels of previous breastfeeding experience among university students in other societies tend to be consistent with the initiation rates of BF in these societies [19]. Undergraduate students intending to breastfeed have exhibited more favourable views, have experienced BF themselves, or were acquainted with someone who has breastfed [20,21]. The BF education module was found to significantly influence middle school students’ attitudes and knowledge regarding BF [22]. Similarly, a BF education program was reported to enhance nursing students’ understanding and positive attitudes towards BF [23]. Investigating young adults’ attitudes is a crucial area of research; the results have indicated that a positive attitude among university undergraduate students towards BF strongly forecasts the desire to breastfeed in both genders [24].

Arab women may provide significant culture-related insights into the determinants influencing BF intentions and infant-feeding decisions in the Arab area, including Syria, where a substantial correlation between intention and the Breastfeeding Behaviour Questionnaire (BBQ) score was demonstrated [13].

This article provides different perspectives on the BF practices of Syria and Hungary. With its rich history and tradition, Syria offers a glimpse into the deeply ingrained cultural practices surrounding BF, as BF often extends beyond mere nutrition. It is related to religious reasons since BF is discussed in the Holy Quran with the statement that breastmilk is the perfect nutrient for the baby [25]. The Holy Quran states that women must breastfeed their children for two whole years [26]. On the other hand, there is a mix of modern and traditional influences in Hungary, with breastfeeding rates having changed over time due to shifting customs, social norms, and healthcare policies. The Ministry of Human Resources supports breastfeeding by promoting flexible and responsive practices in its guidelines [27]. The Hungarian recommendations aim to support mothers with professional and modern assistance to overcome BF challenges, enhancing the percentage of exclusively breastfed infants until around six months [17].

Through this comparative lens, we aim to shed light on the diverse factors that shape BF behaviours in these two societies. By understanding the traditions and sociocultural effects, as well as the challenging contexts, we can glean insights into the barriers and facilitators of BF to understand the effects of all these factors. These factors were chosen to be studied among university students in these two societies; the perspective of young women in society, especially those educated, represents a crucial demographic for understanding BF knowledge. Educated women always have higher BF indicators compared to women with no formal education [12,28].

## 2. Materials and Methods

### 2.1. Participants

This survey was implemented in Syria (in Damascus) and Hungary (in Budapest). The first part was conducted in October and November of 2022 at Damascus University; the sample comprised 317 female students. The second part of the survey was carried out in Budapest, Hungary, in April and May 2023. The Hungarian sample included 303 students from Semmelweis University and Eötvös Loránd University.

This survey was available only in paper format in the two countries’ native languages: Arabic and Hungarian. Independent official translators translated the survey from English to Arabic and Hungarian with a back translation method.

### 2.2. Study Design

This research is part of an extensive survey using a multi-section questionnaire with three modules. This research investigates the behaviours measured by the BBQ developed in 1992 by M. Kay Libbus [29]. This module includes 12 scenarios (Table 1) describing a woman making decisions in specific situations. Participants were required to read the scenarios and choose the most appropriate response from a 6-point Likert scale, ranging from “strongly disagree” to “strongly agree”. No “not sure” or neutral responses were provided; participants had to indicate their agreement or disagreement. There was no correct or wrong answer; we aimed to explore the participants’ behaviours about BF (the Breastfeeding Behaviour Questionnaire (BBQ) can be found in the Supplementary Materials).

### 2.3. Sociodemographic Characteristics of the Samples

This study employed a questionnaire to gather data on sociodemographic characteristics, including nationality, gender, year of birth, father’s education level, mother’s education level, marital status, place of permanent residency, and wealth index. Participants’ current educational level was also documented. The samples from the two countries were nearly balanced regarding nationality, with 51.0% Syrian and 49.0% Hungarian respondents. All students were female, and 58.7% were from medical faculties; most of them (68.3%) were bachelor’s students, while 25.0% were master’s students, and only 6% were PhD students. Concerning parental education, 44.7% of students reported that at least one parent had completed university-level studies. In total, 73.0% of students were residing in urban areas. Marital status showed that 27.0% were married, and 64.0% of the students had a wealth index at an acceptable level.

### 2.4. Measurements

The methodology for this study utilised statistical analysis with the Statistical Package for the Social Sciences (SPSS 25.0) software. Data analysis was carried out using Predictive Analysis Software (PASW 18, formerly known as SPSS). Statistical procedures were completed at a significance level of 5%. Descriptive statistics were performed for demographic variables, scale scores, and the responses to all statements within each scale. Firstly, an ANOVA analysis was applied to show how respondents ranked by nationality distribution when giving their opinions on different statements. A factor analysis was then used to detect latent effects along the 12 variables.

The resulting factors were used to examine the relationship and impact between nationality and the factors using linear regression. Alternatively, a cluster analysis was conducted to detect groups of respondents with different activities.

## 3. Results

### 3.1. Agreement and Disagreement Among Participants in the Total Sample

The highest percentage of disagreement among both nationalities was found in the scenario that discusses the husband’s influence on BF behaviour; regarding the husband’s expression of a preference toward formula feeding due to finding BF embarrassing, it was observed that 94.9% of respondents disagreed with the wife’s decision who decided to bottle-feed instead of BF. Only 5.5% supported this decision change (Table 1).

Some disagreement could be observed among respondents in three other statements. More than 70% (74.1%) of respondents disagreed with a woman’s decision to feed her baby instead of BF based on the belief that formula milk is as good as breast milk. Slightly fewer but still 71.1% of respondents disagreed with choosing formula feeding for the next child due to difficulties with a previous BF experience, such as losing the baby’s weight. When contemplating the reintegration of breastfeeding mothers into the workforce, 69.5% of respondents believed that selecting bottle-feeding for the first child is inappropriate, while 30.5% concurred that opting for formula feeding is justified (Table 1).

An increasing support for other scenarios could be observed in the study. The percentage of opponents and supporters was almost the same for three scenarios. Altogether, 54.6% of the respondents agreed that if a mother wants to breastfeed her baby, even in a restaurant, while wearing her blouse, she should find a private place for this activity. In the following scenario, a similar situation was described for the respondents, but in this case, the presence of a group of friends was included in the scene; 72.9% of the respondents agreed that if it is uncomfortable for the mother’s friends, it is better to withdraw to the car to continue BF. Regarding the scenario of visiting neighbours while a mother is BF her baby in the living room, 56.9% of respondents thought she should stop BF in the presence of visitors. More than half of respondents felt that a mother should take the baby out of the church to breastfeed. As many as 88.2% of respondents thought it was a good decision for a mother to breastfeed despite family history. The highest level of agreement (95.2%) was with the statement in the scenario assessing the doctor’s influence: a pregnant woman who has initially planned to formula feed decides to breastfeed instead due to her doctor’s advice (Table 1).

### 3.2. Differences Between Participants

One of the differences appeared in the first two scenarios, which ask women for the availability of BF in the presence of a female or male. In both situations, there were significant differences between respondents’ views of the two nationalities. Hungarian respondents generally had significantly more positive opinions about the continuation of BF in the case of a female visitor than Syrian respondents. In the case of neighbour visits, when respondents had to decide whether to continue BF in the presence of a male and a female, it could be seen that mainly Syrian respondents had the attitude that the mother should stop BF for this visit. Therefore, the same trend could be observed for both statements, but the association rate (Eta) and the explained variance rate were much higher for the second statement. This indicates that nationality has a significant effect on both but especially in the case of BF inhibition in the presence of both men and women (Table 2).

Moving to the scenarios concerning religious settings, in both cases, Hungarians emphasised that it was more appropriate for women not only to cover the baby or go to the bathroom during BF but also to leave the church. The agreement for these two scenarios among Hungarian participants was 80.3% and 67.4%, respectively, whereas in the Syrian context, responses to questions concerning religious settings display a balanced distribution of agreement and disagreement among both Christian and Muslim Syrian participants. Both ANOVA tests revealed a significant correlation, and the Eta value showed a moderate strength relation in both cases. However, when examining the Eta Squared values, it could be observed that although there was a significant relation, the variance explanation was low, so it can be assumed that other factors also influence the opinions (Table 3).

### 3.3. Factor Analysis

The attitudes behind the statements were also confirmed through factor analysis. The KMO value of the test was 0.689, which made our set of variables susceptible to factor analysis. In addition, the significance level was 0.000, which confirmed the validity of the analysis even further, as the correlation matrix between the statements was significantly different from the null matrix. Using principal component analysis (Table 4), well-differentiated factors could be created. Four principal components were obtained when the latent structure between variables was examined beyond simple descriptive statistics.

By applying Varimax rotation, it became evident which variables belonged to each factor. Four variables represented a pragmatic approach to feeding decisions within the first factor. Situations in which women make decisions about BF and bottle-feeding for different motivations were highlighted in this factor. In doing so, members of the cluster mainly supported the bottle-feeding option. The second factor primarily reflected attitudes toward the acceptability of BF in public places, changes in BF plans, and family history as an influencing indicator. Therefore, this factor examined how decisions regarding BF are made, whether based on public perception or the opinions of family members. The third factor concerned the variables that examined the behavioural patterns that emerge when withdrawal from BF is described in public due to feeling uncomfortable in the situation. The fourth factor included variables that reflected environmental and social pressures on BF decisions (Table 4).

By examining the causality of each sociodemographic variable (gender, marital status, place of residence, wealth, mother’s and father’s level of education) on the principal components, the effects of different attitudes towards BF can be understood. There was no significant relation between the demographic variables and the principal components, but ethnicity significantly impacted the factors.

A significant connection (Sig.: 0.000) between the first factor and nationality could be detected. There was a positive coefficient effect (0.497), which means that the pragmatic approach described by the factor was more strongly present among Hungarian than Syrian students. In contrast to the Syrian respondents, Hungarian respondents were more likely to opt for bottle-feeding, considering motivations and different circumstances. When the second factor was also examined, a significant relationship and a noticeably substantial effect of nationality on the model were observed (Sig.: 0.000). The Beta value (0.341) was slightly weaker, but it can still be stated that nationality influences parametric decisions. In the case of Syrian participants, pragmatic considerations were less important for their decisions. In comparison, Hungarians were more likely to include different opinions or even the degree of acceptability of BF in public places when reporting their decision-making process. Concerning the third factor, nationality had a significant effect (Sig.: 0.000) but still a weak Beta coefficient (0.182), which means that it had a lower impact on the behavioural patterns that emerge during public BF and the necessity to withdraw to avoid unpleasant situations. In this case, Hungarian women were more likely to withdraw or change their behaviour if they were in a public BF situation to prevent uncomfortable situations. Syrian women were less concerned about these aspects. Although Beta had a weak value of 0.142, it was statistically significant (Sig.: 0.000), meaning that nationality had a limited effect on the fourth factor. Regarding environmental and social pressures, it was also apparent that Hungarians were mainly affected by these factors. In the case of Syrian women, it was also noticeable that external factors had little influence on their BF decisions.

### 3.4. Attitude Clusters

Synthesising the previously presented and analysed samples, a K-Means, non-hierarchical cluster analysis procedure was performed to isolate the groups determining the population’s attitudes towards BF. Our results show that respondents can be divided into four homogeneous groups. The distribution of respondents by clusters was as follows. A quarter of the respondents (24.5%) belonged to the cluster of those who opted for External Pressure Choosers. Most of them (31.7%) were Dedicated BF Supporters. In addition, 21.2% of respondents belonged to the cluster of Uncertain BF Supporters and 22.6% to the Pragmatic Bottle-Feeding Supporters.

**Figure 1:**
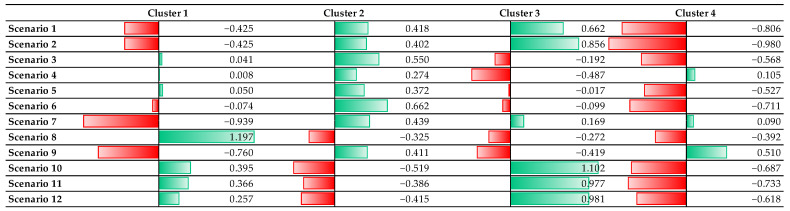
Components of the attitude clusters. (Red: "Negative (minus) values"; Green: "Positive (plus) values").

The cluster of (1) Uncertain BF Supporters was a group of people who were strongly influenced by past negative experiences of BF and thus preferred bottle-feeding to BF. Whether it was the family experience or the spouse’s opinion, they were more likely to opt for a change in plans and choose bottle-feeding over BF. Regarding BF in public places, cluster members considered it better to withdraw in such cases and continue BF in a private place. Alternatively, they supported the suspension of BF at home in certain cases (Figure 1).

The second cluster of (2) Dedicated BF Supporters consisted of respondents who appeared no longer subject to environmental and social pressures. They were not influenced by past feeding experiences or family history and insisted on BF. For situations in public places, they had broadly similar views to those in the first cluster. Thus, they felt that a BF mother in a public place must go outside or retreat to a private place with her child to avoid awkward situations. With regards to BF in a home environment, they were in favour of BF in the company of a woman, but with a man being present, they felt it appropriate to suspend BF (Figure 1).

The term (3) Pragmatic Bottle-Feeding Supporters was used for the third cluster, including those who would choose bottle-feeding for pragmatic reasons. Such reasons could be the need to return to work as soon as possible, previous negative experiences, or information received from others. However, it is interesting to note that BF, as advised by a doctor, was less likely to be accepted if bottle-feeding was already the primary method of choice. Concerning BF in public, it was not believed necessary to retreat to a private place or be concerned about disturbing others while breastfeeding. They also had a positive view of BF at home in the company of a female guest (Figure 1).

Respondents in the fourth cluster were labelled (4) External Pressure Choosers because a doctor’s opinion or family experience would make them change their original intention. Their own experience or intention to work did not influence their decision. They did not even take their husband’s uneasy feelings into account in this regard. When breastfeeding in church, they would not take their child to a private place or outside. If, on the other hand, their circle of friends would be embarrassed by BF in public, they would withdraw for the time of BF. If there were no discomfort around them, they would not feel the need to withdraw unless they had a male visitor at home while breastfeeding (Figure 1).

The data by nationality showed that Syrian respondents mainly belonged to the External Pressure Chooser cluster, and Pragmatic Bottle-Feeding Supporters accounted for only 6% of Syrian respondents. In the case of Hungarians, it could be observed that the Pragmatic Bottle-Feeding Supporter cluster tended to be the most dominant, while the External Pressure Chooser cluster was the most marginal in this nationality (Table 5, Sig: 0.000; Cramer’s V: 0.552).

In the context of the distribution by level of education, most BSc students were in the second cluster, although there was a high percentage of students in the fourth cluster. The least in number were the Uncertain BF Supporters. When students at the MSc level were examined, their majority was also observed in the second cluster. However, there was a substantially lower percentage of respondents in the fourth cluster. Of the PhD students, 36.1% were in the Uncertain BF Supporter cluster and only 19.4% in the Dedicated BF Supporter cluster (Table 6, Sig.: 0.006; Cramer’s V: 0.123).

Respondents could also be separated according to the education level of their fathers and the cluster to which they belonged. Those whose fathers had a university degree were the majority (27.3% and 27.3%) of those in the first two clusters, while the least were the Pragmatic Bottle-Feeding Supporters, who accounted for 18.5%. Respondents with a lower education level of the father were primarily in the second cluster (35.2%) and the least in the first cluster.

In the distinction by place of residence, those who lived in an urban environment were the most likely to belong to the second and fourth clusters (28.2% and 26.9%). The least likely were the Pragmatic decision makers. Among those living in rural areas, Uncertain BF Supporters and External Pressure Chooser clusters were detectable.

Marriage also had an effect on cluster membership. Almost half of the already-married respondents (46.8%) belonged to the fourth cluster, while the largest proportion of unmarried respondents (33.5%) belonged to the second cluster.

## 4. Discussion

This study illustrates BF behaviours among Syrian and Hungarian undergraduate female students. Syria is a Middle Eastern country with one of the lowest rates of exclusive BF in the world [13], while Hungary is an Eastern European country with moderate BF rates [17].

The WHO strongly advocates for BF as the optimal source of nutrition for infants and young children [1]. However, regional differences in BF behaviours indicate that some areas have more barriers than others [2]. Low-income and middle-income countries have longer BF durations than high-income countries [4,9]. Moreover, within the same society, mothers from poorer families in low- to middle-income countries tend to breastfeed more than those from wealthier families, likely due to cost-saving benefits [4].

The findings of the present study suggest that the effect of culture in Syrian society is more pronounced than in Hungarian society. The statistics clearly show that cultural influences play a much larger role in shaping society in Syria compared to Hungary. BF, if there is a female in the room, is not as positively valued by Syrian women as it is by Hungarian women; similarly, Syrian participants are more supportive of the discontinuation of BF if a male is present, while Hungarians are less supportive.

The factor analysis identified four main factors influencing breastfeeding decisions: pragmatism in feeding choice, public breastfeeding, suggestions from a family member and others, as well as private preference to avoid discomfort and environmental/social pressure. The factor of nationality has a highly significant influence on the probability of these factors, as it has a strong and significant effect on each of them. It can be seen that Hungarians are more likely to bottle-feed and have articulated a willingness to accept breastfeeding in public places. Oppositely, it is the Syrian women who will most probably practice public breastfeeding, even if it is uncomfortable. Furthermore, external pressures seem to have more impact on Hungarian women. Thus, our regression analysis denotes the cultural and social differences between the two nationalities.

Syrian and Hungarian participants agree with BF in public places using a BF cover. It is noticeable that, in general, there is a concord in the responses among the participants, making the approximate percentages consistent with other international research which found restrictive attitudes toward exposure to the breast [30,31,32], considering it unacceptable behaviour and to be kept private [32,33].

Following the factor analysis, the cluster analysis identified distinct attitudinal groupings that further lend more concrete meaning to the sociocultural dynamics between the two nationalities. While Syrians mainly were in the External Pressure Chooser cluster, showing a significant influence to family and medical advice, Hungarians were mostly Pragmatic Bottle-Feeding Supporters, highlighting personal convenience and work-related challenges. This distribution is influenced by differences in practical considerations and familial expectations, which appear to be distinct between Hungary and Syria due to cultural and social norms.

These statistics can be explained by the fact that Syrian society has the same concepts of privacy toward BF as the Hungarian, and it has a similar percentage of BF under cover; accordingly, it seems that BF standards in public have become more open and reflect the values of more developed countries.

BF is deeply rooted in Islam’s beliefs and values, which play a crucial role in enhancing health education and boosting exclusive BF rates [34]. In our study, traditions and religious beliefs have a more powerful effect on Syrians than on Hungarians, suggesting a deeper resonance and adherence to religious norms and practices in Syrian society, which is consistent with previous studies that showed religious people to be more opposed to public breastfeeding [35]. In contrast, the religious aspects do not affect the Hungarian’s decisions, taking into consideration that the religious places in Syria are divided according to gender, which adds more aspects of complexity, shaping societal interactions and norms in distinctive ways.

Moreover, the demographics accentuate the above findings because urban individuals are overrepresented in both these clusters (Dedicated Breastfeeding Supporters and External Pressure Choosers), suggesting they are exposed to tremendous societal pressures. Those whose fathers had attained a university-level education were more likely to belong to more supportive clusters, which emphasises the catalytic role of education in developing progressive breastfeeding attitudes.

The high percentage of agreement or disagreement in specific questions reflects a remarkable level of awareness, responsibility, and understanding among participants regarding BF, with slight differences in the consensus on using bottle-feeding as a primary choice. The influence of physicians, family, and partners affects the preference for BF or bottle-feeding in many cases and the possibility of changing BF decisions according to their desires. The influence of social support plays a positive role in the BF decision; support from close friends and the mother’s mother is most important, as previous articles have confirmed [36,37,38]. Breastfeeding mothers need family, friends, colleagues, and community support because it is crucial for increasing exclusive BF rates, and this agrees clearly with previous studies [38].

Similarly, husbands are the ones who have to help mothers the most when it comes to caring for and feeding their babies; their attitude and knowledge are essential in this regard. Husbands can boost the possibility of BF [39] because a well-educated father may encourage his wife and support her more about beginning and maintaining BF [40]. Moreover, the partner’s or wife’s personal standards of exclusive BF could change if she observes the father’s attitude, as the father’s position is considered the most significant in a family, as prior studies have shown [38,41]. On the other hand, the most common reason for preferring bottle-feeding and reducing BF rates is the mother’s feelings about the father’s attitude [42].

Marital status also plays a significant role, with married respondents more frequently appearing in the External Pressure Chooser cluster. This underscores the reinforcing role of spousal relationships in breastfeeding decisions, contrasting with unmarried respondents who are less affected by external commitments.

Most physicians, paediatrics, general practice, and hospital staff workers have a positive attitude toward BF and an encouraging effect on breastfeeding mothers, which has been clearly confirmed before [43,44], while some of them have a neutral position toward exclusive BF [45]. Doctors often have limited awareness of the practical aspects of aiding a breastfeeding mother in overcoming challenges related to BF [46], with few exceptions. Unfortunately, doctors frequently give breastfeeding women incorrect information about BF, which can cause premature weaning [47].

BF is the best nutrient for infants [1,9], and the use of baby formulas and bottles increases the risk of oral diseases and tooth decay [48]; it can observed that a bottle-feeding preference among students is sometimes due to a lack of sufficient information or working mothers.

A busy schedule for nursing mothers is a crucial challenge, whether she is a working mother or a stay-at-home mother, adding additional responsibilities to her lifestyle [49]. An unsupportive spouse of the family is one of the biggest challenges stay-at-home and working nursing mothers face [50,51], while additional pressure could appear in the working mother’s life such as an unsupportive work environment [50,52]; feeling guilty is also a common dilemma working mothers have to cope with when they leave their infant to return to work [50]. Returning to work was recognised as one of the most frequent challenges for not exclusively BF or not continuing BF for 24 months [53,54]; BF in the workplace is not always an available choice for breastfeeding mothers [54], and it has been well-known that unemployed mothers are more likely to practice full BF compared to employed mothers [55], even though they want to and have positive attitudes toward BF in both Arab and European countries [53,54], which is in contrast with our study which shows that a high percentage of students (70%) do not agree with depending on formula exclusively when going back to work, in addition to the feeling of embarrassment about BF in their workplace, which reduces the rate of exclusive breastfeeding [53]. The most critical point of not breastfeeding in the workplace is related to the maternity leave period; in our study, half of the Syrian students expressed positive behaviour toward continuing BF if the mother returned to work, while only 14% of Hungarians had the same perspective. These percentages are inconsistent with the Syrian and Hungarian governmental rules about maternal leaves. The Syrian Labor Law No. 17 of 2010 stipulates that a female worker is allowed to have a fully paid maternity leave of 120 days for the first child, 90 days for the second, and 75 days for the third child; these short periods in Syria are similar to the practices in many other Arab countries [55,56], but this discrepancy could be attributed to many factors, including Syrian cultural traditions prioritising BF in any circumstances, socioeconomic factors influencing access to formula, or perhaps a deeper understanding of Syrian society on nutritional and developmental BF benefits.

By contrast, working parents receive paid maternity and paternity leave according to the Hungarian system. With at least two weeks being obligatory, new mothers are entitled to 24 weeks of paid maternity leave—longer than the Organization for Economic Cooperation and Development countries’ average (18 weeks) [57]. Nevertheless, a critical point must be taken into consideration, namely, Hungarian families’ ability to choose formula for their infant, which they can purchase with a medical prescription for half-price during the first six months of the child’s life [58]—during the same 24 weeks of paid parental leave.

In general, myths about BF are widespread worldwide, and they affect the BF decision; however, it can be observed that in our study, the students had not been affected by these myths. It is a fact that the amount of fat, not the alveoli that produce milk, determines the size of the breast. Regardless of the mother’s breast size, the breast tissue required to feed an infant increases throughout pregnancy. In reality, mothers with smaller breasts can still produce enough milk to maintain a sufficient supply if they recognise their infants’ requirements and breastfeed when necessary [47].

Another myth related to BF is “the not satisfied baby”, which is a worldwide concept among breastfeeding women and their parents and close family, and it is a famous reason for stopping BF or complementing it with bottle-feeding. Weaning breastfed newborns to infant formula is expected since it is mistakenly believed that the baby’s continuing crying is an indication of hunger [59]; although the duration of BF of the second child is significantly related to previous BF experience, many mothers complement BF with formula due to wrong concepts or lack of information about BF [60].

Regression analysis confirms that nationality has a measurable impact on attitudes across all factors. Syrian respondents show a stronger adherence to familial and traditional influences, while Hungarians are more pragmatic, with external pressures disproportionately affecting their decisions. This underlines how cultural and societal structures inform personal choices regarding breastfeeding.

Finally, regarding the clusters identified in this study, there are differences between the clusters by nationality, education, and place of residence. By nationality, Syrian respondents are mainly in the External Pressure Chooser cluster. In contrast, most Hungarians belong to the Pragmatic Bottle-Feeding Supporters group, which suggests that Hungarians are more influenced by pragmatic considerations than Syrians.

Those having a father with a university degree are more likely to be found in the clusters of Uncertain and Dedicated Breastfeeding Supporters, showing that it is mainly the father’s university degree that has a supportive effect on attitudes. The Dedicated Breastfeeding Supporters and External Pressure Choosers clusters are especially high among urban residents, suggesting that social influences may be more frequent in urban settings. Concerning marital status, the proportions show that spousal relationships are more robust against external factors, while if someone is not married, they are less influenced by a stronger personal commitment.

## 5. Strengths and Limitations

The strength of this study lies in the comparison between Syria and Hungary, which sheds light on how their cultural, ethical, and traditional differences impact breastfeeding behaviours. The specific demographic feature of this study, involving university students only, provides a good opportunity to explore the perception of breastfeeding among future mothers. Additionally, using the BBQ adds reliability to this study because it is a standard questionnaire that measures participant behaviours toward breastfeeding, which helps to compare results and reduce misinterpretation. However, the sample may be considered minor and not representative of the whole population in either country, since university students’ education level or backgrounds could affect breastfeeding behaviours in the questionnaire. Furthermore, the data reported may have been biased by social desirability, especially in topics with a unique character, such as public breastfeeding, breastfeeding in formal places, and pressures from the family. Also, due to the study design, it is impossible to determine causality; we can interpret associations but not causations between sociocultural factors or nationality and breastfeeding behaviours. Finally, because the questionnaire was translated, the slight differences in language could affect comprehension and response accuracy, potentially impacting the reliability of the data.

## 6. Conclusions

When the results from Syria are compared and analysed with those from Hungary, observable differences and similarities appear, shedding light on the distinct sociocultural backgrounds. This comparative study emphasises the diverse factors influencing BF practices in these two societies and allows us to grasp the socioeconomic contexts of BF.

This study highlights specific gaps in the knowledge and behaviour primarily related to BF in public and in front of visitors; BF in religious spaces; the influence of physicians, extended family, and partners on the final decision regarding BF; the effects of breast shape on the BF decision; insufficient milk supply; and the ease and practicality of BF for a working mother.

Targeting young, educated students is good for understanding the challenges and improving awareness since BF knowledge has also been positively associated with previous BF experience.

By understanding the factors that support breastfeeding, we can suggest strategies to improve breastfeeding practices in different frames. Supportive environmental conditions in work and universities need to be provided to help working and student mothers; in addition, public health campaigns can be aimed at normalising breastfeeding in public using private tools.

The data show that social factors are essential in shaping both societies’ attitudes, behaviours, and norms. These data can be used in further studies, public health programs, and university settings. The findings of this study are valuable for multiple stakeholders, including public health students, healthcare professionals, public health policymakers, and any organization working on maternal and child health initiatives in Syria and Hungary.

## Figures and Tables

**Table 1:** Statistical results for Scenarios 1 and 12 of the BBQ among Syrian and Hungarian participants (%, N = 620).

Scenario	Very Strongly Disagree	Strongly Disagree	Disagree	Agree	Strongly Agree	Very Strongly Agree
**Scen. 1**: Jane Johnson, a new mother, is breastfeeding her baby in the living room. Her girlfriend from next door comes to see the new baby. Jane covers her breast and the baby’s head with a receiving blanket and the baby continues to nurse while the two women talk.	4.9	5.1	7.9	23.6	21.3	37.2
**Scen. 2**: Estelle Green is breastfeeding her baby in the living room. The man and woman from next door come to see the new baby. Estelle covers her breast and the baby’s head with a receiving blanket and the baby continues to breastfeed while the neighbours talk.	15.7	12.5	14.9	19.3	14.4	23.1
**Scen. 3**: Martha Smith is at McDonald’s eating lunch with her girlfriends. When her baby wakes up and seems hungry, she decides to breastfeed him under her blouse.	12.3	11.3	22.8	27.3	15.3	11.0
**Scen. 4**: Kathy Brown is eating lunch at Dairy Queen with her girlfriends. When her baby wakes up and seems hungry, she decides to breastfeed him under her blouse. Her friends are embarrassed by this, so she takes him out to the car to breastfeed him instead.	5.1	6.7	15.2	35.9	20.3	16.7
**Scen. 5**: Anne Evans and her husband take their baby to church. When it is time for the baby to breastfeed, Anne takes her into the ladies’ bathroom.	8.2	7.2	19.6	27.3	18.6	19.1
**Scen. 6**: Marie Schultz and her husband take their baby to church. When it is time for the baby to eat, Marie breastfeeds the baby under her blouse. She also covers the baby’s head with a receiving blanket in case the blouse slips.	10.0	10.8	20.3	26.1	17.7	15.1
**Scen. 7**: June Moon is expecting her first baby and wants to breastfeed. June’s mother tells her that no one in their family has been able to successfully breastfeed since all the women have small breasts and can’t make enough milk. June decides to breastfeed anyway.	3.3	2.6	5.9	18.6	21.0	48.6
**Scen. 8**: Laura Baxter is expecting her first baby and wants to breastfeed. Laura’s husband wants her to bottle-feed the baby because he says that breastfeeding is “embarrassing”. Laura decides to bottle-feed instead of breastfeeding.	62.7	19.7	12.2	3.8	1.0	0.7
**Scen. 9**: Linda Martin is pregnant, and her doctor tells her that she should plan to breastfeed her new baby. Linda had planned to bottle-feed but changes her mind.	1.3	1.5	2.0	16.9	22.6	55.7
**Scen. 10**: Jane Blaine, who is expecting her first baby, was advised to breastfeed her new baby because “human milk is better for human babies”. Jane decides to bottle-feed instead because she has heard that formula is every bit as good as breastmilk.	30.6	18.6	24.9	15.5	5.6	4.8
**Scen. 11**: Peggy Kelly is expecting her first baby very soon. She was advised to breastfeed but decides to bottle-feed because she wants to go back to work when the baby is 3 months old and has heard that a breastfed baby won’t take a bottle.	22.1	19.0	28.4	20.3	6.6	3.6
**Scen. 12**: Jeanette James is expecting her second baby. Even though she has been told that breastfeeding is better for babies, she decides to bottle-feed. She tried to breastfeed her first baby and had to stop because the baby lost weight during the first week.	23.1	19.5	28.5	16.2	7.5	5.1

**Table 2:** ANOVA table for Scenarios 1 and 2 of the BBQ (N = 608).

Scenario	Mean			Sig.	Eta	Eta Squared
	Syrian	Hungarian	Total			
Scenario 1	4.17	5.12	4.63	0.000	0.333	0.111
Scenario 2	2.86	4.67	3.73	0.000	0.516	0.266

**Table 3:** ANOVA table for Scenarios 5 and 6 of the BBQ (N=608).

Scenario	Mean			Sig.	Eta	Eta Squared
	Syrian	Hungarian	Total			
Scenario 5	3.44	4.56	3.98	0.000	0.378	0.143
Scenario 6	3.41	4.13	3.76	0.000	0.239	0.057

**Table 4:** Principal component analysis.

	1 Feeding Decision Making	2 Social and Familial Influences on Feeding	3 Public BF Acceptance	4 Environmental Comfort in BF
Scenario 1		0.754		
Scenario 2		0.691		
Scenario 3			0.865	
Scenario 4				0.850
Scenario 5				0.738
Scenario 6			0.812	
Scenario 7		0.655		
Scenario 8		−0.431		
Scenario 9	−0.579			
Scenario 10	0.842			
Scenario 11	0.834			
Scenario 12	0.798			

**Table 5:** Distribution of clusters within nationalities.

		Cluster Number of Case				Total
		Uncertain BF Supporters	Dedicated BF Supporters	Pragmatic Bottle-Feeding Supporters	External Pressure Chooser	
Nationality	Syrian	21.8%	28.4%	6.0%	43.8%	100.0%
	Hungarian	20.8%	35.6%	40.5%	3.2%	100.0%
Total		21.3%	31.8%	22.3%	24.6%	100.0%

**Table 6:** Distribution of clusters within the level of education.

		Cluster Number of Case				Total
		Uncertain Breastfeeding Supporters	Dedicated Breastfeeding Supporters	Pragmatic Bottle-Feeding Supporters	External Pressure Chooser	
Level of education	BSc	18.6%	32.0%	21.0%	28.4%	100.0%
	MSc	25.3%	34.0%	26.0%	14.7%	100.0%
	PhD	36.1%	19.4%	22.2%	22.2%	100.0%
Total		21.3%	31.8%	22.3%	24.6%	100.0%

## Data Availability

The raw data supporting the conclusions of this article will be made available by the authors on request.

## Supplementary Materials

The following supporting information can be downloaded at: https://www.mdpi.com/article/10.3390/nu17020288/s1. The Breastfeeding Behavior Questionnaire (BBQ).
